# Massive release of extracellular vesicles from cancer cells after photodynamic treatment or chemotherapy

**DOI:** 10.1038/srep35376

**Published:** 2016-10-18

**Authors:** Kelly Aubertin, Amanda K. A. Silva, Nathalie Luciani, Ana Espinosa, Aurélie Djemat, Dominique Charue, François Gallet, Olivier Blanc-Brude, Claire Wilhelm

**Affiliations:** 1 Laboratoire Matière et Systèmes Complexes, UMR 7057, CNRS and Université Paris Diderot, 10 rue Alice Domon et Léonie Duquet, 75205 Paris cedex 13, France; 2 Animalerie BUFFON. Institut Jacques Monod. UMR 7592 CNRS - Université Paris Diderot, 75205 PARIS Cedex 13, France; 3 ParCC Paris Cardiovascular Center, INSERM UMRs 970, Université Paris Descartes, PRES Sorbonne-Paris-Cité, et Hôpital Européen Georges Pompidou, Assistance Publique-Hôpitaux de Paris, 56 rue Leblanc, 75908 Paris CEDEX 15, France

## Abstract

Photodynamic therapy is an emerging cancer treatment that is particularly adapted for localized malignant tumor. The phototherapeutic agent is generally injected in the bloodstream and circulates in the whole organism as a chemotherapeutic agent, but needs light triggering to induce localized therapeutic effects. We found that one of the responses of *in vitro* and *in vivo* cancer cells to photodynamic therapy was a massive production and emission of extracellular vesicles (EVs): only 1 hour after the photo-activation, thousands of vesicles per cell were emitted in the extracellular medium. A similar effect has been found after treatment with Doxorubicin (chemotherapy), but far less EVs were produced, even 24 hours after the treatment. Furthermore, we found that the released EVs could transfer extracellular membrane components, drugs and even large intracellular objects to naive target cells. *In vivo*, photodynamic treatment and chemotherapy increased the levels of circulating EVs several fold, confirming the vast induction of cancer cell vesiculation triggered by anti-cancer therapies.

The tumor microenvironment plays an important role in cancer development and progression^1^,^2^. Cancer cells (CCs) can modulate the host environment, early in the neoplastic process, in order to ensure tumor survival and expansion^3^. Complex cellular crosstalk mechanisms orchestrate this process, including immunosuppression^4^,^5^,^6^. Soluble factors such as cytokines act mainly at or near their site of release, and are thus unlikely to account for the multiple systemic immune suppressive phenomena observed in cancer patients^7^. An increasing body of evidence indicates that far-reaching pathways, such as vesicle-mediated cell-cell communication, play a pivotal role in remote tumor cell signaling. The term “rececrine” has been used to describe signaling via cell-released extracellular vesicles (EVs), related to the secretion of active cell receptors, including oncoproteins and growth factor receptors, as well as anchored and loaded signaling molecules^8^. The ability of EVs to convey cellular signals over long distances is related to their double-layer membrane, which protects their biomolecular contents. Such vesicles carry and protect signaling packages and convey messages which stimulate or inhibit cells in an ectopic manner^9^,^10^. They have been found to participate in physiological processes such as organ development^11^ as well as pathological ones such as cancer.

CCs use rececrine signaling to promote immune escape^12^,^13^, invasiveness^14^ and angiogenesis^9^,^15^, as well as to induce an aggressive phenotype^10^ and drug resistance^16^,^17^,^18^. EVs are also able to export cytotoxic drugs such as doxorubicin^19^ and cisplatin^16^ out of the cell, in order to reduce their intracellular drug concentration and to resist to cancer treatment.

Although tumor-cell-derived EVs are now recognized as playing a major role in CC survival and progression, and in the dissemination of detrimental bioactive molecules, little is known about the effect of antitumor treatments on EV release.

Here we show that a photodynamic therapy (Foscan^®^ photosensitizer) and a chemotherapeutic agent (doxorubicin) can trigger massive EV release from CCs. Both antitumor treatments are currently being used in clinical practice^20^,^21^,^22^,^23^. Photodynamic therapy (PDT) generates cytotoxic radical species through interaction between a photosensitizer and oxygen triggered by light activation. PDT is approved for the management of head and neck tumors^24^ and it is at the stage of clinical trial for the treatment of early prostate cancers^25^ and non-melanoma skin cancer^26^. Doxorubicin (DOX), a DNA intercalating drug, is used to treat solid tumors because of its very broad antitumor spectrum^27^. We found that Foscan^^®^^ uptake by cancer cells followed by light exposure induced massive and near-instantaneous vesicle release. This process was less intense and less rapid with DOX, but was still pronounced. We quantified EV release with two complementary methods: flow cytometry and nanoparticles tracking analysis. Worryingly, we found that EV release was higher at intermediate drug concentrations having only moderate cytotoxic efficacy. We then showed that EVs are able to transport cell membrane- or endosome-associated probes, as well as intracellular drugs. EVs were also able to transfer their drug cargo to naive healthy cells *in vitro*, with cytotoxic consequences. *In vivo*, both treatments induced EV release in tumor-bearing model mice. Hence, our multiscale and multimethod study revealed that mild cancer treatments can trigger massive release of noxious EVs. These findings imply that the assessment of the efficacy of a given therapeutic approach must take into account the triangle that interrelates drug concentration, target cell damage and EV release.

## Results

### Cancer cell treatment with Foscan^®^ photosensitizer or Doxorubicin induced a dose-dependent cytotoxic effect

In order to appraise the effect of cytotoxic drugs on CC vesiculation *in vitro*, PC-3 in the entire text prostatic CCs were subjected to PDT (with Foscan^®^) or chemotherapy (with DOX). Foscan^®^ (5,10,15,20-tetra(m-hydroxyphenyl)chlorin), a reduced porphyrin also known as m-THPC, is a well-established cell photosensitizer that has been in clinical use for over 10 years. It is a neutral hydrophobic molecule with a molecular weight of 681 Da, and is able to interact with plasma proteins^28^ and membranes (crossing the latter by passive diffusion^24^,^29^). Doxorubicin (DOX) is an amphoteric drug with a deprotonable phenolic group and a protonable amino group^30^. This DNA intercalating agent is widely used to treat a broad spectrum of cancers^27^.

**Figure 1:**
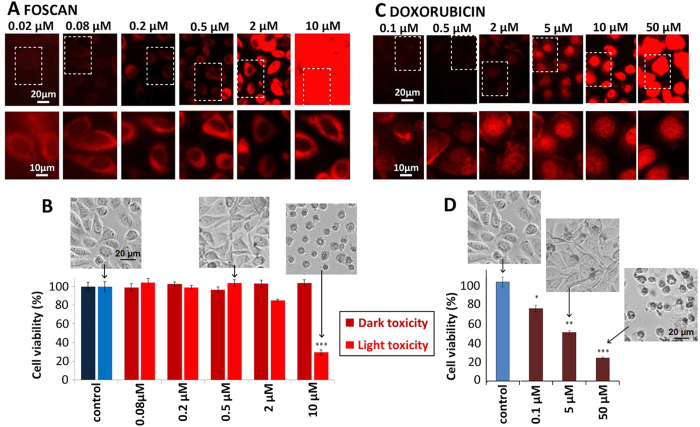
Cancer cell treatment with the Foscan^®^ photosensitizer or Doxorubicin. (**A**) Fluorescence microscopy of CCs 24 hours after 2-hour incubation with Foscan^®^ from 0.02 to 10 μM. Concentration-dependent drug uptake was shown by the enhancement of direct fluorescence emission as the concentration increased. All images were acquired with the same acquisition time (20 ms). The same scaling was applied to the whole images, while images shown in the inset were autoscaled. Diffuse cytoplasmic distribution was observed in all conditions. (**B**) Cell viability was assessed by measuring metabolic activity, either in the absence of light exposure (dark toxicity) or 24 hours following light exposure (light toxicity). No metabolic activity changes were observed except for 2 and 10 μM concentrations. (**C**) Fluorescence microscopy of CCs 24 hours following 2-hour incubation with DOX from 0.1 to 50 μM. Concentration-dependent drug uptake was shown by direct fluorescence emission (top panel with the same scaling, for the same exposure time of 200 ms). A higher magnification is provided in the autoscaled bottom panel, where DOX was mainly contained in the nucleus. (**D**) Cell viability was assessed by measuring metabolic activity 24 h after DOX incubation, and ranged from 70% to 20% at 0.1 μM and 50 μM, respectively.

For PDT, CCs were incubated with Foscan^®^ photosensitizer at concentrations from 0.02 to 10 μM for 2 hours (Fig. 1A). Foscan^®^ cell loading increased with the extracellular concentration, and Foscan^®^ was distributed throughout the cytoplasm, in keeping with its hydrophobicity^24^. The cells were then exposed to light, their viability (Fig. 1B) was assessed via a metabolic activity assay 48 hours following drug incubation and 24 hours following light exposure (light toxicity) and compared to ‘dark’ toxicity (drug internalization without light). Dark toxicity was minimal, while light exposure reduced viability to 80% and 30% for 2 μM and 10 μM Foscan^®^, respectively.

For chemotherapy, CCs were incubated with DOX from 0.1 to 50 μM for 2 hours (Fig. 1C). DOX intracellular uptake was concentration-dependent, and was mainly localized in the nucleus, regardless of extracellular concentration, with slight cytoplasmic distribution. DOX localization in the nucleus was related to its affinity for DNA, in keeping with its DNA-intercalating action^31^. Metabolic activity was determined via enzymatic activity, evaluated by Alamar blue test. In contrast to Foscan^®^, DOX induced cell damage directly, without additional trigger. Cell viability was affected more strongly by DOX than Foscan^®^-PDT (Fig. 1D), dropping to 70% at 0.1 μM and 20% at 50 μM.

### Chemotherapy and photodynamic therapy induce extracellular vesicle release *in vitro*

**Figure 2:**
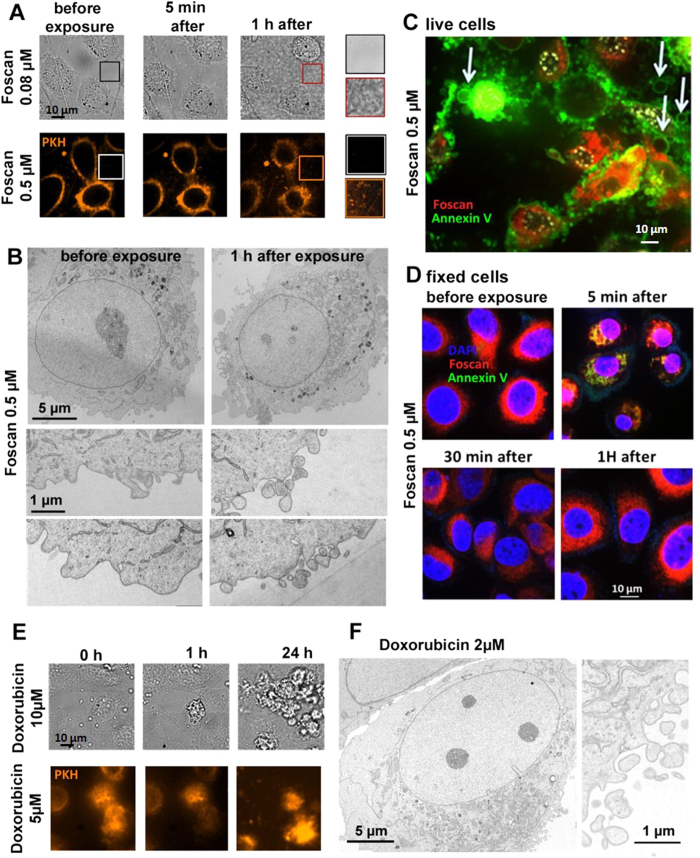
Vesicle release from CCs following PDT or DOX. (**A**) Optical microscopy of living cells before and after Foscan^®^ PDT (0.08 μM in bright field images and 0.5 μM in the fluorescence images, with PKH orange staining of the cell membranes). Vesiculation was observed in the culture medium, which became cloudy/milky, as observed in bright field images. Under orange fluorescence, bright and highly mobile spots appeared outside the cells (see movie S1). (**B**) Transmission electron micrographs of cells loaded with Foscan^®^ (0.5 μM), before (left) or 1 hour after (right) light exposure. Intense extracellular vesicle shedding occurred 1 hour after PDT. (**C**) Fluorescence imaging of living Foscan^®^-loaded (0.5 μM) CCs 5 min after light exposure. Cell membrane is annexin positive for most of the cells, indicating apoptosis. Apoptosis is further evidenced by the shedding of vesicles. Annexin-positive EVs are indicated by white arrows. (**D**) Analysis of CC apoptosis by fluorescence microscopy of annexin-A5 staining in fixed cells, before exposure, 5 min, 30 min and 1 hour after PDT (Foscan^®^ 0.5 μM), as compared to a control without light exposure. Red fluorescence is emitted by Foscan^®^, and the apoptosis marker annexin-A5-FITC appears in green. Transient annexin-A5 staining was observed only 5 min after exposure. The apoptosis marker was no longer detected 30 min or 1 hour later, indicating a reversible effect. (**E**) Bright-field and fluorescence (orange membrane staining) images before and after DOX treatment (10 and 5 μM, respectively). Vesicles were observed in the extracellular medium, but only after 24 hours (see orange spots in the 5 μM condition), and they were less abundant than after PDT. (**F**) Transmission electron micrographs of CCs 24 hours after DOX exposure (2 μM). At 2 μM, the cells shed EVs, but still fewer than after PDT.

Immediately after light-exposure for PDT, the culture medium became cloudy, due to the presence of abundant objects subject to Brownian motion (Fig. 2A, Supplementary Fig. 1, Supplementary Movie 1). A high level of these Brownian objects could be visualized at 1 hour time point after light exposure (Supplementary Fig. 2, Supplementary Movie 2). This was observed in both bright-field and fluorescence images (CC membranes were stained orange with PKH just before light exposure) (Supplementary Fig. 3, Supplementary Movie 3). This effect was ascribed to the release of EVs, as the Brownian objects were membrane-stained in the same way as their parent cells. EV release was further confirmed by transmission electron microscopy (TEM) (Fig. 2B). EV release is generally associated with early apoptotic events, and one hallmark of EVs is positive staining for annexin-A5^32^, due to phosphatidylserine translocation from the inner to the outer leaflet of the cell membrane. In order to investigate phosphatidylserine translocation, we stained living CCs for annexin-A5, after incubation with 0.5 μM Foscan^®^ and 5 min after light exposure. After the photodynamic insult, annexin-A5-labeled vesicles were found to be released by annexin-A5-positive cells, indicating that they carry apoptotic markers from their parent cells (Fig. 2C). Time-dependent laser observation of annexin-A5 binding to living cells is tricky, however, because laser exposure can itself trigger apoptosis. We therefore examined annexin-A5 binding as a function of time after light exposure, using fixed cells. Transient annexin staining was observed immediately after PDT (Fig. 2D). Annexin-A5 bound to the cells 5 min after light exposure, confirming our observations in living cells. However, the cells were already negative for annexin-A5 30 min after light exposure, and were still negative after 1 hour, when abundant vesicle release is obvious. These findings revealed an early, transient and reversible apoptotic response that was probably accompanied by concurrent release of microvesicles enriched in annexin-A5.

DOX also induced EV release, as demonstrated by optical microscopy and TEM (Fig. 2E,F, respectively), albeit to a lesser extent than PDT. As DOX does not require an external trigger, vesiculation was investigated immediately after drug incubation. Real-time fluorescence microscopy (Fig. 2E) of CCs after membrane staining with PKH showed almost no vesicle release 1 hour after drug incubation. After 24 hours, some mobile orange vesicles were detected in the cells vicinity. On TEM images (Fig. 2F, 24-h) both 2 and 50 μM DOX induced vesicle formation at the plasma membrane, but the 50 μM concentration was associated with additional nuclear fragmentation, as observed previously^33^,^34^.

### Photodynamic therapy induced higher vesicle shedding then chemotherapy according to nanoparticle tracking analysis (NTA) and flow cytometry

**Figure 3:**
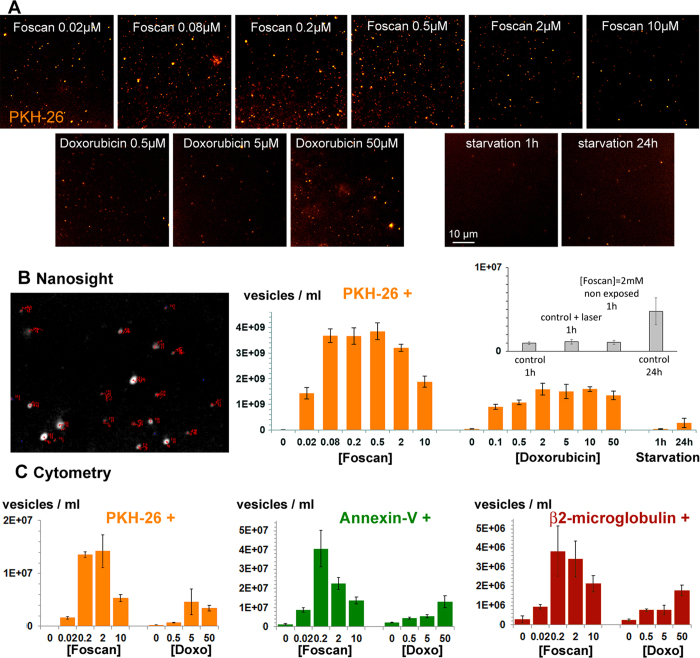
Vesicle quantification by NTA and FACS. (**A**) Fluorescence microscopy of conditioned medium after PDT, DOX treatment or starvation of PKH-stained CCs. A large number of fluorescent submicronic events were observed, particularly at the intermediate Foscan^®^ concentration. (**B**) Quantification of EV release (per million cells) by NTA in all conditions. Quantification was based on the detection of a PKH-26-stained population. Vesicle release was more abundant at intermediate Foscan^®^ concentrations, creating a bell-shaped curve, while it increased slightly as the DOX concentration rose. Note that 0 mM corresponds to 1-hour starvation (left) or 24-hour starvation (right). The inset corresponds to unlabelled/unexposed; labeled/unexposed or unlabeled/exposed controls (**C**) Quantification of EV release (EV/ml) by FACS. Quantification was based on the detection of populations positive for PKH-26, annexin-A5 or human β2-microglobulin. The concentration-response profile was in keeping with the results of NTA, for all the fluorescence markers.

Vesicle shedding was measured after PDT and chemotherapy. The incubation media were collected 1 hour and 24 hour after treatment, respectively, for all concentrations (Foscan^®^ 0.02 to 10 μM, DOX 0.1 to 50 μM). Starvation-induced vesicle release was also quantified after 24 hour in serum-free medium. Controls were incubated for 1 hour in complete medium. The conditioned media were first observed by fluorescence microscopy, which revealed numerous orange spot-like vesicles (Fig. 3A). Interestingly, EV release was maximal at intermediate Foscan^®^ concentrations (range 0.2–2 μM). DOX induced fewer vesicles, and their numbers did not increase with drug concentration.

These observations were confirmed by quantitative Nanoparticle tracking analysis (NTA) (Fig. 3B, Supplementary Movies 4 and 5, Supplementary Figs 4 and 5) and flow cytometry (Fig. 3C) analyses of incubation media, and expressed as vesicle concentrations. NTA is a recent method that provides the size and the concentration of vesicles by tracking their Brownian motion (here in the fluorescence channel). In control conditions (unlabeled and unexposed cells), there were only 10^7 ^EVs/ml release within 1 hour. The same range was obtained for labeled/unexposed or unlabeled/exposed cells (Fig. 3B inset and Supplementary Fig. 6). No vesicles were detected in medium only. By contrast, PDT induced 4 × 10^9 ^EVs/ml release within 1 hour at the intermediate Foscan^®^ concentration, i.e. ~400-fold increase over controls. 24-hour starvation led to only 3 × 10^8 ^vesicles/ml, 15 times less than with PDT after 1 hour. Importantly, for PDT, EV release was not proportional to Foscan^®^ concentration: vesicle shedding rose with drug concentration up to a maximum between 0.5 and 2 μM, and then declined gradually. Hence, there was a statistically significant difference (p < 0.0001) between EV release at 0.5 and 10 μM Foscan^®^, while it was similar at 10 μM and 0.1 μM. Finally, DOX induced about 2 × 10^9 ^vesicles/ml in 24-hours, a value 30 times higher than seen in control conditions, and 6 times higher than in starvation conditions. Regardless the triggering stimuli, vesicle size was in 300–400 nm range, according to NTA (Supplementary Figs 6 and 7).

FACS (Fig. 3C) is the high-throughput, multi-parametric method most commonly used to quantify EVs, providing concentration, and fluorescence detection. We used FACS to quantify vesicles stained with PKH, annexin-A5 and β2 microglobulin (gating and counting data are provided in Supplementary Fig. 8). The EV release profiles as a function of the Foscan^®^ or DOX concentration indicated that vesicles labeled with PKH (which stains vesicle membranes), annexin-A5 and human β2 microglobulin (a marker specific of human CCs) were similar.

NTA gave values about 100-fold higher than FACS, as NTA can detect smaller vesicles that conventional FACS, with a detection limit of about 300 nm^35^. The limited sensitivity and resolution of flow cytometers is a concern^36^. It has been reported once that FACS could underestimate vesicle concentrations by almost 300-fold compared to NTA^37^.

### EVs transport endosome-associated materials (nanoparticles) as well as intracellular drug, and transfer their contents to naïve cells

**Figure 4:**
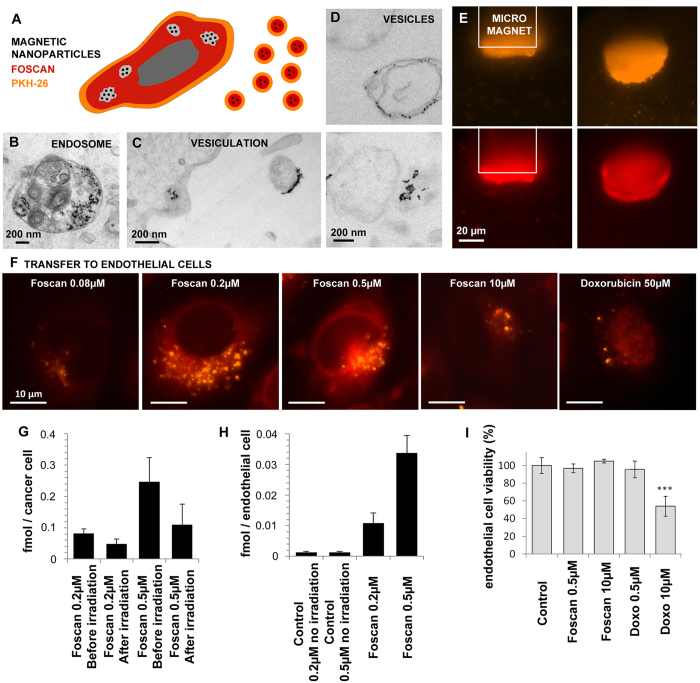
Vesicle contents and their transfer to naïve endothelial cells. (**A**) Schematic representation of treatment-induced release of vesicles carrying magnetic nanoprobes (black spots), PKH dye (orange) and Foscan^®^ (red). (**B**) Magnetic nanoprobes, visualized as electron-dense spots, were initially located in the endosomal compartment of CCs. (**C**,**D**) 1 hour after PDT (light exposure), the magnetic nanoprobes were contained in the released vesicles. (**E**) Vesicles released by CCs loaded with magnetic nanoprobes were further tested for their magnetism: they were attracted with a 50-μm-diameter cylindrical micromagnet while simultaneously emitting red and orange fluorescence, reflecting co-encapsulation of the magnetic probes and PKH (top) or Foscan^®^ (bottom). (**F**) Fluorescence micrographs of naïve ECs following incubation with conditioned medium from CCs exposed to DOX or PDT. Endothelial recipient cells displayed characteristic PKH and Foscan^®^ or DOX fluorescence emission, indicating their uptake of vesicles containing the drug. (**G**) The Foscan^®^ intracellular concentration in CCs decreased after light exposure, suggesting a partial loss of their Foscan^®^ content via vesiculation. (**H**) Foscan^®^ was detected in recipient ECs (right). Concentration-dependent transfer was observed: recipient ECs contained a larger drug load when incubated with conditioned medium from CCs treated with Foscan^®^ at 0.5 μM than at 0.2 μM. Foscan^®^ was scarcely detected in recipient ECs incubated with conditioned medium from Foscan^®^-treated but non-irradiated cells. (**I**) Recipient ECs showed no reduction in viability after incubation with conditioned medium from CCs (no dark toxicity). Conversely, a cytotoxic effect was observed during incubation in conditioned medium from cells treated with DOX, which does not require a physical trigger to exert its effects.

This abundant EV release suggests that anticancer treatments themselves might induce the spreading of oncogenes and oncoproteins, and that drug release from EVs might induce drug resistance at remote sites. We therefore examined whether EVs contained the drug to which their parent cells had been exposed (Fig. 4A), in conditions of maximal EV release, i.e. after Foscan^®^-PDT.

We first investigated the subcellular origin of EVs by using magnetic nanoprobes. Electron micrographs of cancer cells (CCs) showed that they internalized the magnetic nanoprobes within endosomes/lysosomes (Fig. 4B)^38^. Following PDT, magnetic nanoprobes were found to be contained in released EVs (Fig. 4C,D). The conditioned medium was then analyzed in a set-up^39^ designed to measure EV attraction by a micro-magnet (Fig. 4E). The magnet attracted submicronic vesicles emitting both PKH orange and Foscan^®^ red fluorescences.

We then examined whether the EVs could transfer their drug cargo to naïve endothelial cells (ECs). ECs were incubated with the conditioned media and observed by fluorescence microscopy. Both the orange marker (PKH, spots inside endosomes) and red fluorescence (photosensitizer or DOX) were clearly visible inside the cells (Fig. 4F, Supplementary Figs 9–11). Interestingly, both drugs were redistributed to their cytoplasmic (photosensitizer) or nuclear (DOX) locations. Remarkably, the higher the vesicle density, the more intense their endothelial cell uptake (compare Fig. 4F with Fig. 3B). Fluorescence spectrophotometry showed that PDT significantly reduced the amount of Foscan^®^ inside CCs (Fig. 4G). The photosensitizer fluorescence was recovered in recipient ECs (Fig. 4H), indicating drug transfer. Of note, ECs incubated with medium conditioned by CCs treated with Foscan^®^ 0.2 or 0.5 μM, without light exposure, displayed no fluorescence, confirming that EV-mediated drug transfer required photodynamic stimulation. Finally, as expected, EV-mediated Foscan^®^ transfer had no cytotoxic effect on the recipient ECs, as they were not irradiated. In contrast, EC viability was negatively impacted by DOX transfer (Fig. 4I).

These data indicate that PDT and DOX induce the release of vesicles carrying plasma membrane constituents and endosome contents. Release of such vesicles might contribute to anticancer drug resistance by reducing the intracellular drug concentration. In addition, it might disseminate oncogenes or oncoproteins that could affect healthy cells at remote locations.

### PDT and chemotherapy induced vesicle release *in vivo*

**Figure 5:**
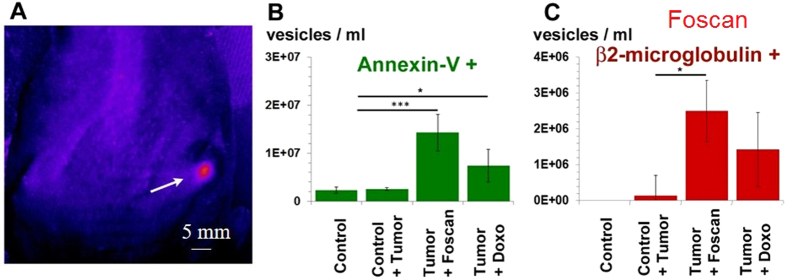
PDT and DOX induction of vesicle release *in vivo* (mice with subcutaneous PC3 tumors). (**A**) Fluorescence imaging of a Foscan^®^-injected mouse, showing the presence of the drug at the tumor site. (**B**) FACS of plasma from mice treated with DOX or PDT showed more annexin-A5-positive vesicles than in healthy controls and untreated tumor-bearing mice. (**C**) Vesicles released after DOX or PDT were human β2-microglobulin-positive, indicating that they originated from the human CCs.

In order to investigate EV shedding *in vivo*, PC3 tumor-bearing mice were injected with DOX (1.5 mM) or Foscan^®^ (150 μM), then analyzed by fluorescence imaging. Characteristic Foscan^®^ fluorescence was detected in the tumor region, showing the presence of the drug (Fig. 5A). Mice were then light-irradiated 24 hours after Foscan^®^ injection. Blood was collected 24 hours after DOX or light exposure, and plasma was analyzed for EVs by FACS. The concentration of annexin-A5-positive EVs was 3- and 6-fold higher following DOX and PDT, respectively, as compared to healthy control mice and untreated tumor-bearing mice (Fig. 5B). A similar trend was observed for human β2-microglobulin-positive EVs, indicating that they originated from the human CCs (Fig. 5C).

In summary, both antitumor therapies induced the vast release of EVs carrying CC material (drug, oncogenes, proteins, etc.) into the bloodstream, and these EVs could be taken up by neighboring as well as distant healthy cells.

## Discussion

EV release can be both constitutive and stimulus-triggered. In particular, EV shedding can be induced by cell activation or stress^40^. As shown here, in the first quantitative study of its type, cytotoxic insult stimulated EV shedding, especially following PDT at sub-lethal doses. By comparison, starvation (for 24 hours) led to far less abundant vesicle release, which was 15 times lower than the peak reached within 1 hour after PDT. EV emission after PDT was not only the most abundant, but also extremely rapid. The bell-shaped EV release curve as a function of the Foscan^®^ concentration (Fig. 3B,C) is very informative. It supports the hypothesis that a mild photodynamic insult triggers reversible apoptosis and major EV release, whereas a strong photosensitizer insult induces irreversible cell death, possibly directly through cell necrosis, without triggering such a large vesicle release.

These results suggest that “mild” PDT may have multiple drawbacks in terms of treatment failure and EV release, in a worst-case scenario. Indeed, EV release would propagate cancer signaling molecules such as oncoproteins and oncogenic transcripts that might contribute to horizontal transformation and phenotypic reprogramming of recipient cells. For instance, it has been reported that EVs can convey the oncogenic form (EGFRvIII) of the epidermal growth factor receptor from aggressive to indolent CCs, increasing their capacity for anchorage-independent growth^10^. EVs can also harbor tumor DNA sequences and mediate their horizontal transfer to non-malignant cells^41^. EVs released from CCs can promote the transformation of normal fibroblasts and epithelial cells, conferring enhanced survival capability and anchorage-independent growth^42^. In a related example, EV-mediated transfer of oncoproteins may promote metastasis by “educating” bone marrow progenitors to support the constitution of pre-metastatic niches that shelter upcoming melanoma cells^43^. To the best of our knowledge, we provide the first *in vitro* evidence that sub-lethal PDT may lead to abundant EV release. Together, these data support the hypothesis that abundant EV release triggered by mild cytotoxic regimen may in fact worsen the outcome of cancer patients.

We also show that EVs can inherit membrane markers, drugs, and endosomal contents from parent cells. Previous studies showed that EVs could transfer cytotoxic drugs such as DOX and cisplatin to the extracellular medium^16^,^19^,^44^. However, these studies failed to demonstrate that drug treatment itself triggered EV release. The quantitative relationship between drug concentration and EV release had not previously been investigated. We also provide the first evidence that EVs released after PDT or DOX exposure can convey a drug cargo to naïve healthy cells, with cytotoxic consequences.

These observations raise the issue of the impact of anti-tumor therapy on vesicle release *in vivo*. Some studies have suggested that effective treatment strategies reduce EV emission. Shao *et al*. investigated EVs as biomarkers of glioblastoma treatment efficacy. They studied glioblastoma vesiculation in response to treatment with either alkylating agents (temozolomide) or heat shock protein inhibitors (HSP90-targeted geldanamycin). They tested two concentrations of each drug and found lower EV release with the higher concentrations, which was attributed to drug-induced cell death^45^. A decrease in EV concentration in the urine of prostate cancer patients was reported during androgen deprivation therapy (ADT). Despite wide variations in exosome concentrations among healthy subjects and patients, three months of ADT induced a ~2-fold decrease in the patients’ exosome levels^46^. Mege *et al*. equally report that the circulating levels of EVs depend on the cancer evolution, remission being associated to reduced circulating EVs near to healthy levels^47^.

Our *in vivo* experiments indicate that DOX and Foscan^®^ PDT increase the level of circulating EVs. This stimulation combined with the tumoral origin of the circulating EVs raises severe concerns about the iatrogenic and unexpected dissemination of drugs, oncogenes and oncoproteins. EV release, *in vivo* and *in vitro*, was more potent in response to PDT than chemotherapy. EV-induced photosensitizer dissemination could notably aggravate cutaneous photosensitivity. Propagation of oncomolecules mediated by PDT-induced vesiculation could induce cell transformation and modulate the tumor microenvironment. For instance, PDT has been found to indirectly promote tumor invasiveness^48^, increase tumoral VEGF expression^49^ and induce changes in the microenvironment via metalloproteinases^50^. Circulating tumor-derived EVs are prone to interact with the stroma and both healthy and cancer tissues, in proximal and distant sites. The released EVs may thus counterbalance the desired regional limitation of the treatment, and may represent an underestimated source of adverse effects during PDT.

We show that antitumoral treatment, and especially PDT, can induce massive release of EVs. This unsuspected effect could well neutralize the intended topical and tissue specificity of the treatment, and might ultimately influence tumor progression and outcome. Indeed, we found that EVs carry apoptosis markers, drugs from their parent cells, tumor membrane components, and endosome contents. We found that EVs transferred their drug cargo to naïve recipient cells *in vitro*, with “second-hand” cytotoxic effects. In tumor-bearing mice, the same antitumor treatments induced EV release. These findings therefore suggest that cancer treatment monitoring should include the quantification of circulating tumor EVs, as these may affect the ultimate efficacy and tolerability of, for example, chemotherapy and PDT.

## Methods

### Cell culture and labeling

Human prostatic cancer cells (PC-3, ATCC ^®^ CRL-1435™) were cultured in T75 flasks at 37 °C with 5% CO_2_ in Dulbeco’s modified Eagle’s medium (DMEM) completed with 10% fetal bovine serum and 1% penicillin-streptomycin. The cells were brought to confluence in glass-bottomed 10-mm Petri dishes for high-magnification immersion microscopy. The cells were labelled with annexin-A5-FITC or PKH 26 Fluorescent Cell Linker as recommended by the manufacturer (Sigma).

### Drug treatment

Foscan^®^ m-THPC (5,10,15,20-tetra(3-hydroxyphenyl)chlorin) was purchased from Frontier Scientific. Stock solutions were prepared in ethanol, handled in the dark, and stored at −20 °C. PC3 cells seeded in 10 cm^2^ dishes were incubated in RPMI culture medium containing m-THPC at 0.02, 0.08, 0.2, 0.5, 2 or 10 μM at 37 °C for 2 hours. After incubation, the cells were rinsed three times with RPMI and placed in complete DMEM for an overnight chase. The day after, m-THPC-labeled cells were exposed to light for 5 s at a wavelength of 470 nm (7.5 J/cm^2^) under a microscope (DMIRB Leica; Leica Microsystems, Wetzlar, Germany). DOX (Sigma Aldrich) stock solutions at 50 mg/mL in water were stored at −20 °C. PC3 cells were incubated with complete DMEM containing DOX at 0.1, 0.5, 2, 5, 10 and 50 μM at 37 °C for 2 hours. After incubation, the cells were rinsed three times with RPMI and placed in complete DMEM for 24 hours. In order to access cytotoxic effects, Alamar Blue (Invitrogen) was used according to the supplier’s instructions, 24 hours after light exposure or drug incubation (PDT or DOX, respectively). For the starvation control, PC3 cells were washed and placed in serum-free RPMI for 24 hours.

### EV quantification by nanoparticle tracking analysis (NTA)

PC3 cell conditioned medium following PDT, DOX or starvation was analysed with a NanoSight LM10 instrument (NanoSight, Amesbury, UK) for EV quantification. A monochromatic laser beam at 532 nm was applied to the sample. A high band pass filter at 565 nm was used to analyse PKH-labelled events only. NTA post-acquisition settings were optimized and kept constant during triplicate analyses, and each video was analysed with NTA software to obtain concentration data.

### EV quantification by flow cytometry (FACS)

Phosphatidylserine-positive (PS+) EVs were quantified as previously described^51^,^52^. Briefly, EVs were labeled with 2 μl FITC-conjugated annexin-A5 (Roche Diagnostics, France) diluted in 100 μl reaction buffer with 5 mM CaCl2. CaCl2 was omitted in negative controls. EVs were analyzed on a LSR-II flow cytometer (BD Biosciences) and identified in forward light scatter (FSc) and side-angle light scatter (SSc) intensity dot plots set at logarithmic gain, as events of 0.1–1 μm in diameter. EV-size events were then analyzed in fluorescence dot plots to determine annexin-A5 labeling. Absolute EV concentrations were determined with respect to calibrated fluorescent microbeads (Flowcount™; Beckman Coulter). In order to determine cellular origin, some EV were double labeled with mouse PE-coupled anti-human β2-macroglobulin antibody. Positive staining was established against a matched isotype IgG analyzed in parallel.

### Magnetic probe internalization and magnetophoresis

The 0.5-μM m-THPC-labeled cells were incubated for 30 min with magnetic nanoprobes (maghemite, negatively charged, 8 nm diameter, iron concentration 2 mM) one day before light exposure. Following PDT, the conditioned medium was collected and subjected to a magnetic field gradient created by a micromagnet^39^. Briefly, the device consists of a glass slide/coverslip chamber integrating a 50-μm-diameter nickel rod submitted to a 150-mT uniform magnetic field from a rectangular magnet positioned aside. The trajectories of EVs heading towards the micromagnet tip, indicative of magnetic nanoprobe encapsulation, were observed with a 63X oil objective on an optical microscope (DMIRB Leica; Leica Microsystems, Germany) connected to a CCD camera and a computer. The covering of the magnetic tip with vesicles can also be detected in fluorescence to colocalize a fluorescent marker with the magnetic nanoparticles.

### Transmission electron microscopy

PC3 cells were fixed in 5% glutaraldehyde in 0.1 mol/L sodium cacodylate buffer, before treatment, 1 hour after PDT, and 24 hours after DOX treament. The samples were gradually dehydrated in ethanol and stained with osmium tetroxide containing 1.5% potassium cyanoferrate. After Epon embedding, samples were sectioned (70 nm) and observed with a Zeiss EM902 electron microscope operating at 80 keV (GABI, INRA - MIMA2-MET).

### Confocal imaging

Cells were observed with an Olympus JX81/BX61 device/Yokogawa CSU device spinning-disk microscope (Andor Technology plc, Northern Ireland), equipped with a 63X oil objective (Olympus). Cells were fixed with 4% PFA at room temperature for 15 min. For m-THPC and DAPI, the excitation wavelength was 405 nm, and fluorescence emission was collected with filters at 685 and 445 nm, respectively. Annexin-A5-FITC excitation was carried out at 488 nm and fluorescence emission was collected using a filter at 525 nm. Image J software was used to process the images.

### Animal experiments

Research procedure was carried out in accordance with the European guidelines and French national guidelines for the protection of animals used in a research, issued by the Ministry of Agriculture. All animal experiments were approved by Buffon ethics committee (Université Paris Diderot). Six-week-old NMRI male nude mice provided by Janvier Laboratories France were injected subcutaneously with 2 × 10^6^ PC-3 human prostate carcinoma cells in 100 μL of physiological saline, in the left and right flanks. When the solid tumors reached about 5 mm in diameter, the animals were randomized into three groups (m-THPC-injected, DOX-injected and untreated controls). Healthy untreated mice were also studied. In the Foscan^®^ group, animals were irradiated with a 650-nm laser at a fluence of 7 J/cm^2^ (200 mW/cm^2^ for 35 seconds), 24 hours after Foscan^®^ injection. Fluorescence imaging was carried out with an Apogee ALTAU47 CCD camera. Mice were exposed to blue filtered light (400±25 nm). Fluorescence emission was detected above 600 nm by using a high-pass filter. Images were treated with Macbiophotonics ImageJ software. Mouse blood was collected by intracardiac puncture, transferred to heparinized tubes, and centrifuged at 500 *g* for 5 minutes. The supernatant was again centrifuged at 2000 *g* for 15 minutes and the plasma thus obtained was analyzed by FACS.

### Statistics

All data are reported as mean values ± standard deviation (error bars). Student’s t test was used to evaluate significance, with a confidence level of 99% to be considered significant. ***p < 0.001. **p < 0.01. *p < 0.05.

## Additional Information

**How to cite this article**: Aubertin, K. *et al*. Massive release of extracellular vesicles from cancer cells after photodynamic treatment or chemotherapy. *Sci. Rep.* **6**, 35376; doi: 10.1038/srep35376 (2016).

## Supplementary Material

Supplementary Movie 1

Supplementary Movie 2

Supplementary Movie 3

Supplementary Movie 4

Supplementary Movie 5

Supplementary Information
