# Supplementary material for: Massive release of extracellular vesicles from cancer cells after photodynamic treatment or chemotherapy
Source: Sci Rep. 2016 Oct 18;6:35376. doi: 10.1038/srep35376 (PMC5067517; doi:10.1038/srep35376)
Supplement: Supplementary Information [file srep35376-s6.pdf]

SUPPLEMENTARY INFORMATION

Massive release of extracellular vesicles from cancer cells after photodynamic treatment or chemotherapy

Kelly Aubertin[a], Amanda K. A. Silva[a*], Nathalie Luciani[a], Ana Espinosa[a], Aurélie Djemat[b], Dominique Charue[c], François Gallet[a], Olivier Blanc-Brude[c], and Claire Wilhelm[a*]

[a] Laboratoire Matière et Systèmes Complexes, UMR 7057, CNRS and Université Paris Diderot, 10 rue Alice Domon et Léonie Duquet, 75205 Paris cedex 13, France.

[b] Animalerie BUFFON. Institut Jacques Monod. UMR 7592 CNRS - Université Paris Diderot, 75205 PARIS Cedex 13

[c] ParCC Paris Cardiovascular Center, INSERM UMRs 970, Université Paris Descartes, PRES Sorbonne-Paris-Cité, et Hôpital Européen Georges Pompidou, Assistance Publique-Hôpitaux de Paris, 56 rue Leblanc, 75908 Paris CEDEX 15, France.

[*]To whom correspondence should be addressed. Email: amanda.silva@univ-paris-diderot.fr; claire.wilhelm@univ-paris-diderot.fr

1. Supplementary movie legends

Supplementary Movie 1: Onset of vesicle release by PC3 cells (labeled at [Foscan]=2 µM) following photodynamic insult by time-lapse optical microscopy. Cells are initially shown at rest in bright field images. They are then light exposed to induce photodynamic effect (characteristic Foscan fluorescence emission in fluorescence images). The onset of vesicle release is rapidly visualized in bright field images by the observation of numerous objects in the extracellular medium exhibiting enhanced Brownian motions. Movie accelerated 50 times.

Supplementary Movie 2: Time-lapse optical microscopy movie of PC3 cells (labeled at [Foscan]=0.2 µM) 1 hour after photodynamic insult showing a vast amount of EVs moving in a Brownian way in the extracellular culture medium. Movie accelerated 20 times.

Supplementary Movie 3: Time-lapse fluorescence microscopy movie of PC3 cells (labeled at [Foscan=0.5 µM) with membrane PKH-stained 1 hour following photodynamic insult evidencing that the EVs floating in the extracellular culture medium display PKH fluorescence emission inherited from their parent cells. Movie accelerated 100 times.

Supplementary Movie 4: Representative time-lapse movies obtained during analysis of the conditioned extracellular medium by nanoparticle tracking analysis (NTA), using fluorescence mode to analyse PKH-labeled events. The conditioned medium was collected 1 hour after photodynamic insult of PKH-stained PC3 cells previously incubated with Foscan at a concentration ranging from 0.02 to 10 µM. The control (no PDT, thus corresponding to 1 hour starvation) is also shown for comparison. Movie in real time.

Supplementary Movie 5: Representative time-lapse movies obtained during analysis of the conditioned extracellular medium by nanoparticle tracking analysis (NTA), using

fluorescence mode to analyse PKH-labeled events. The conditioned medium of PKH-stained PC3 cells was collected 24 hours following doxorubicin treatment at 0.5 to 50 µM concentration range compared to 24 hours of starvation (no doxorubicin). Movie in real time.

2. Supplementary figures

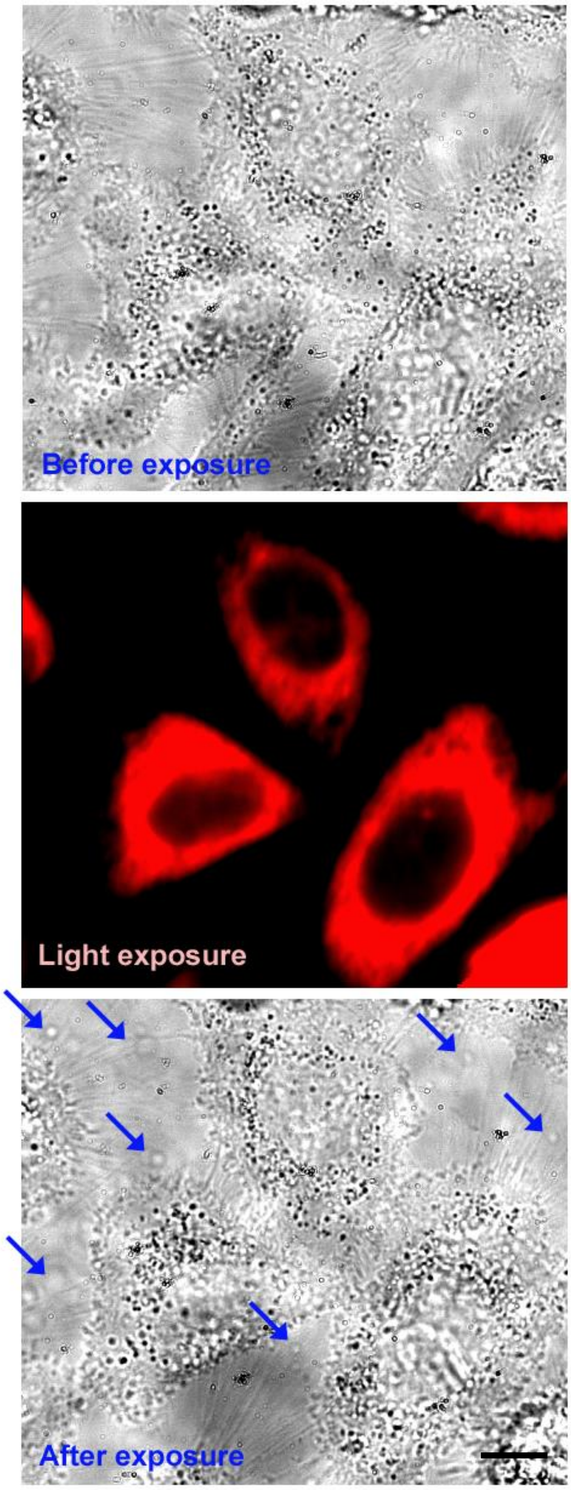

Supplementary Figure 1: Frames from Supplementary Movie 1 showing the onset of vesicle release by PC3 cells (labeled at [Foscan]=2 mM) following photodynamic insult by time-

lapse optical microscopy. Left: cells at rest in a bright field image. Middle: Cells were light exposed to induce photodynamic effect, as evidenced by characteristic Foscan fluorescence emission in a fluorescence image. Right: The onset of vesicle release by light exposed cells is visualized by the observation of Brownian objects freely floating in the conditioned culture medium in a bright field image (blue arrows). Scale bar = 10 µm.

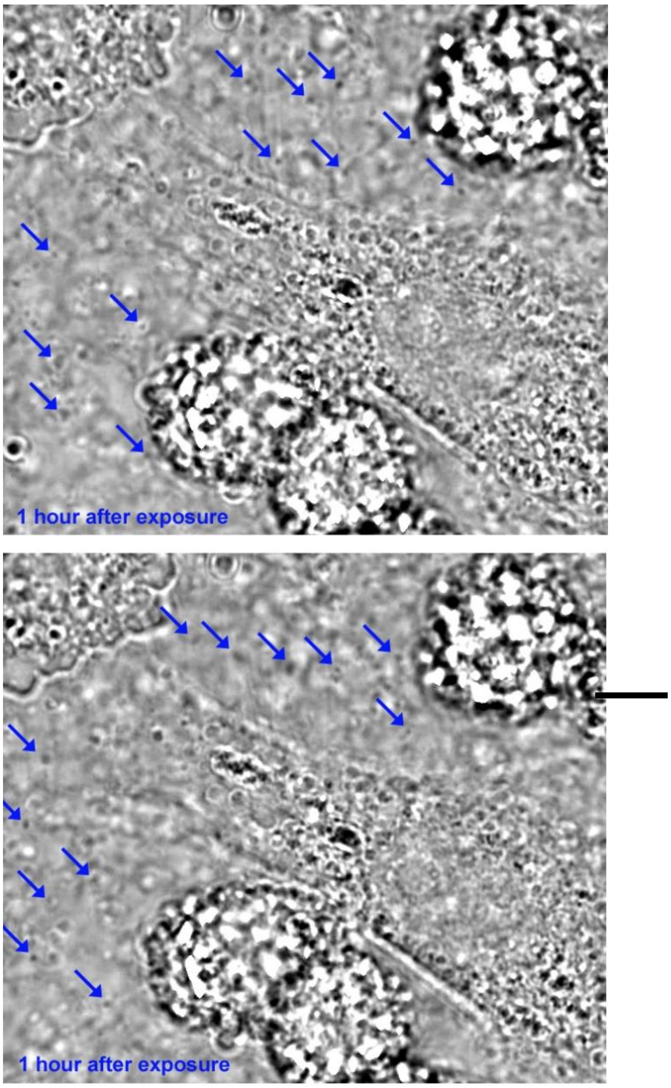

Supplementary Figure 2: Frames from Supplementary Movie 2 showing abundant vesicles (blue arrows identify some of them) in the extracellular medium of PC3 cells (labeled at [Foscan]=0.2 mM) 1 hour after photodynamic insult. Top: first image of the movie; Bottom: last image (40 s after). Scale bar = 10 µm.

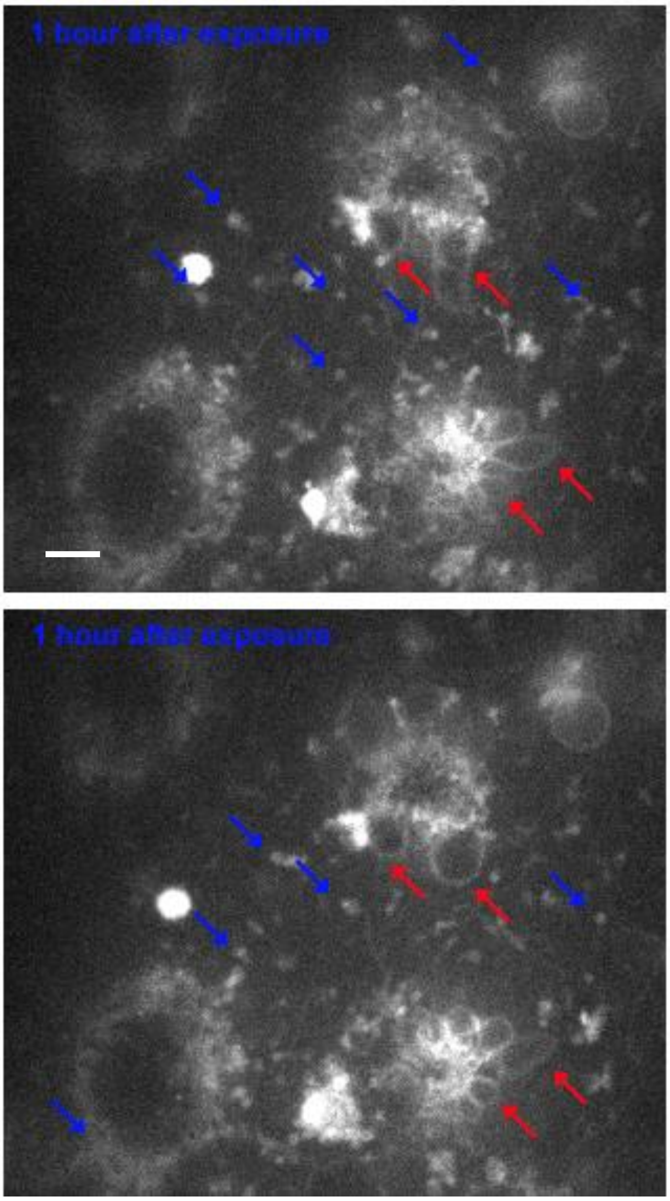

Supplementary Figure 3: Successives frames (2 min apart) extracted from Supplementary Movie 3. The membrane of the PC3 cells (labeled at [Foscan]=0.5 mM) is stained with fluorescent PKH. Vesicles dispersed in the extracellular medium (blue arrows indicate some of them) display the same fluorescence, evidencing their membranous origin. Besides, we can observe in real time EVs formation at the cell membrane (membrane blebbing, red arrows). Scale bar = 10 µm.

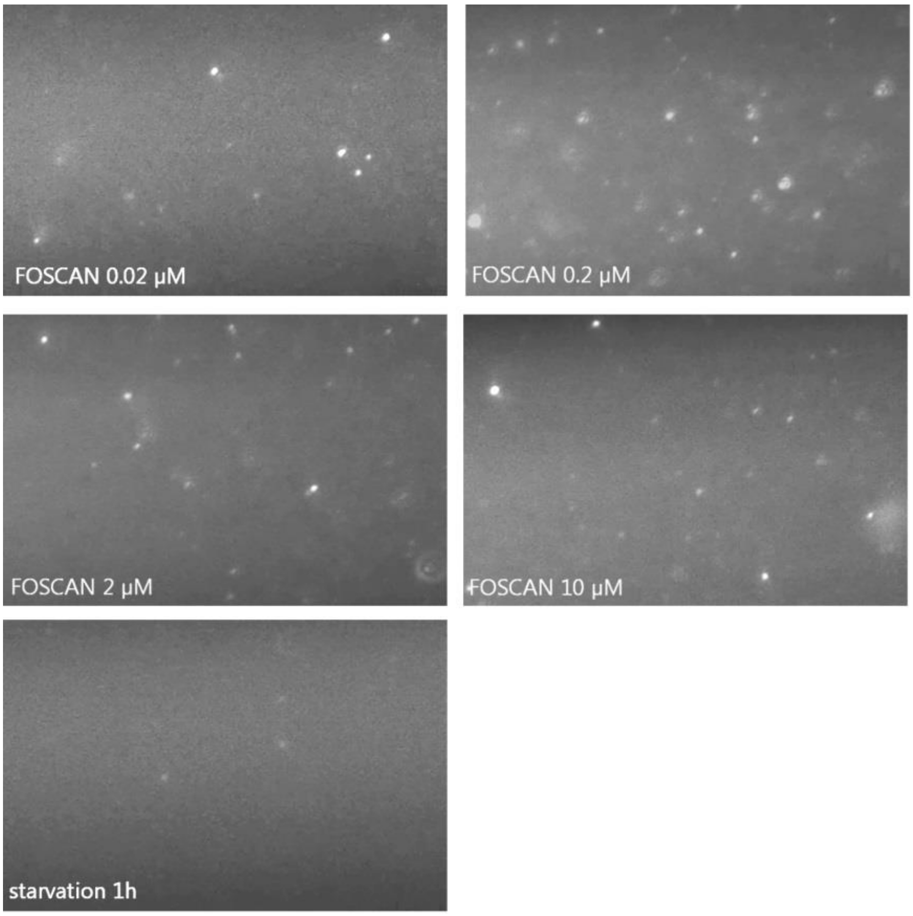

Supplementary Figure 4: Frames from Supplementary Movie 4 of the conditioned culture medium from PC3 cells 1 hour following photodynamic insult obtained by nanoparticle tracking analysis using fluorescence mode to analyse PKH-labelled events only. PC3 cells previously labelled with PKH were incubated with Foscan at a concentration ranging from 0.02 to 10 µM. Vesicle release induced by photodynamic insult was maximal at intermediate Foscan concentrations (0.2 µM) and it was more intense than following 1h of starvation regardless the Foscan concentration.

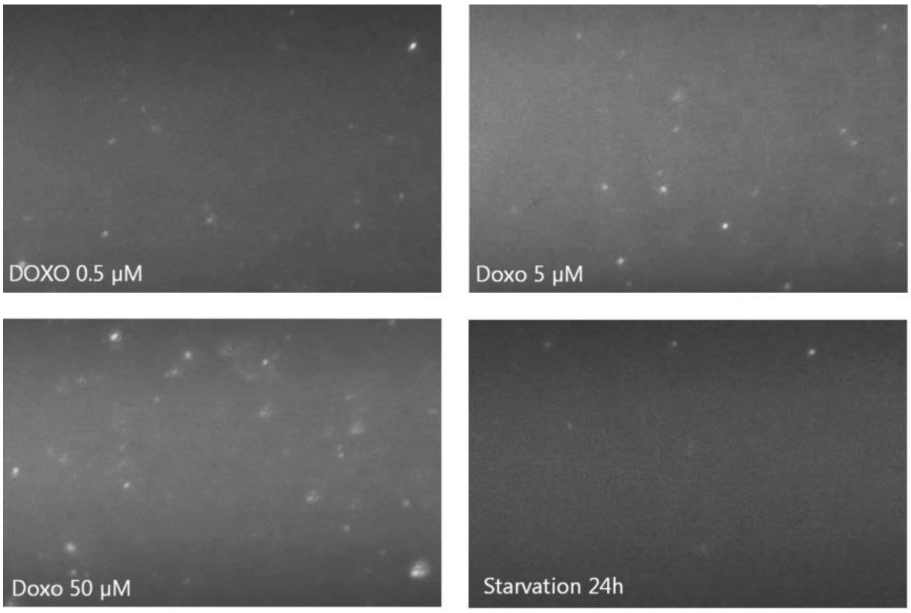

Supplementary Figure 5: Frames from Supplementary Movie 5 of the conditioned culture medium from PC3 cells 1 hour following doxorubicin treatment obtained by nanoparticle tracking analysis using fluorescence mode to analyse PKH-labelled events only. PC3 cells previously labelled with PKH were incubated with doxorubicin at a concentration ranging from 0.5 to 50 µM. Vesicle release induced by doxorubicin was more intense than following 24 h of starvation regardless the doxorubicin concentration.

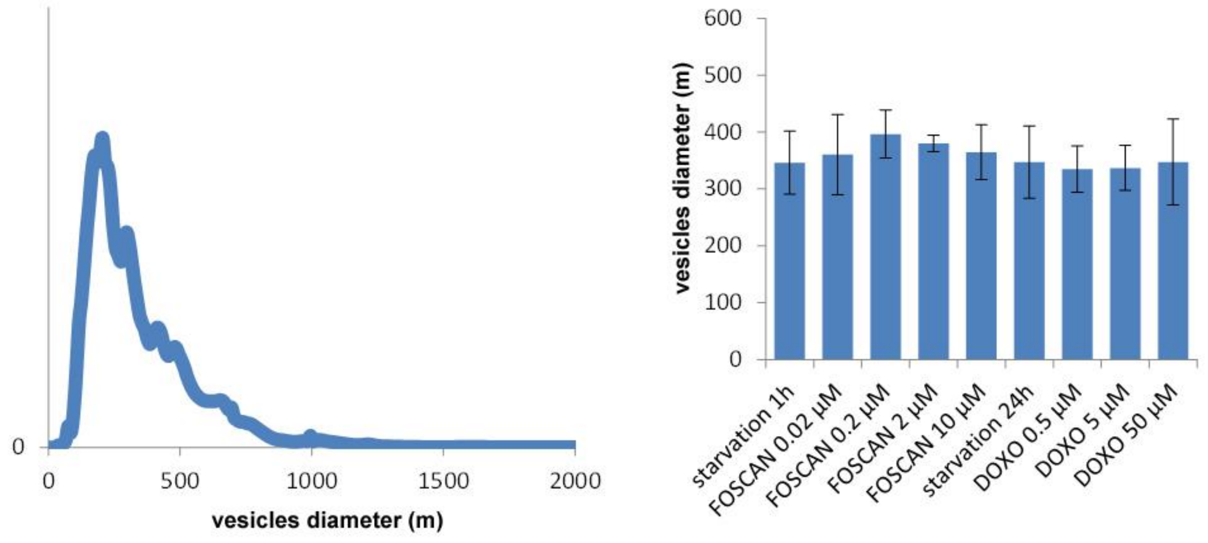

Supplementary Figure 6: Size analysis of vesicles from PC3 cells (previously labelled with PKH) produced in response to starvation, photodynamic insult or doxorubicin treatment obtained by nanoparticle tracking analysis using fluorescence mode to analyse PKH-labelled events only. Size distribution of vesicles following photodynamic insult (Foscan concentration of 0.5µM) (left). Average size of vesicles induced by starvation, photodynamic insult or doxorubicin treatment at different drug concentrations (right).

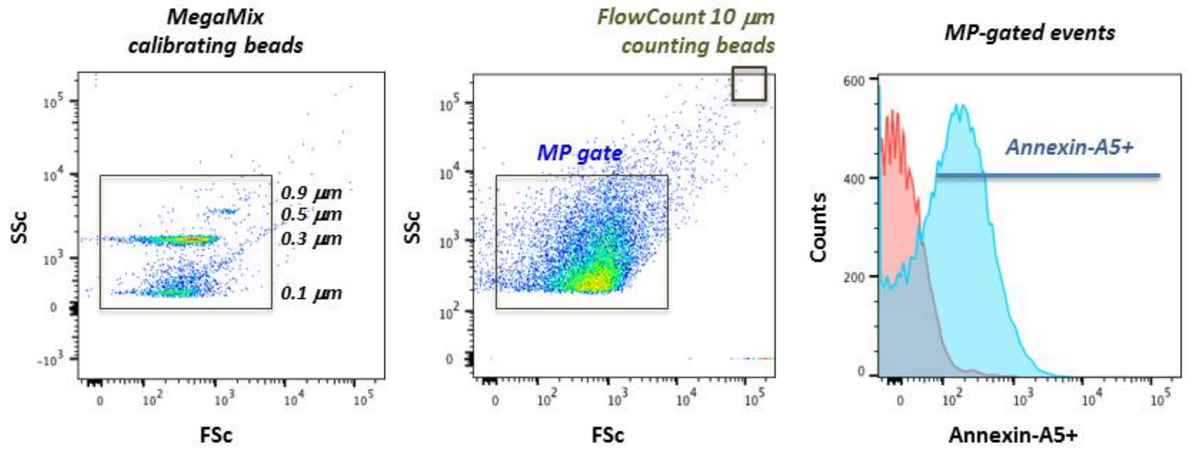

Supplementary Figure 7: Phosphatidylserine-positive (PS+) MP were quantified as previously described using a LSR-II flow cytometer (BD Biosciences). Events of 0.1-1 µm in diameter were plotted in a window (MP gate) set around reference calibrated microbeads (MegaMix™ ; 0.1, 0.3, 0.5, 0.9 µm diameter), identified in forward light scatter (FSc) and side-angle light scatter (SSc) intensity dot plots. MP-size events, labeled with FITC-conjugated annexin-A5 and CaCl2, were analyzed in fluorescence dot plots to determine positive labeling. Absolute MP concentrations were determined with respect to calibrated fluorescent microbeads (FlowCount™; 10 µm diameter). The specificity of the annexin-A5 labeling of MP-size events was determined by including (blue peak) or removing CaCl2 (negative control as red peak).

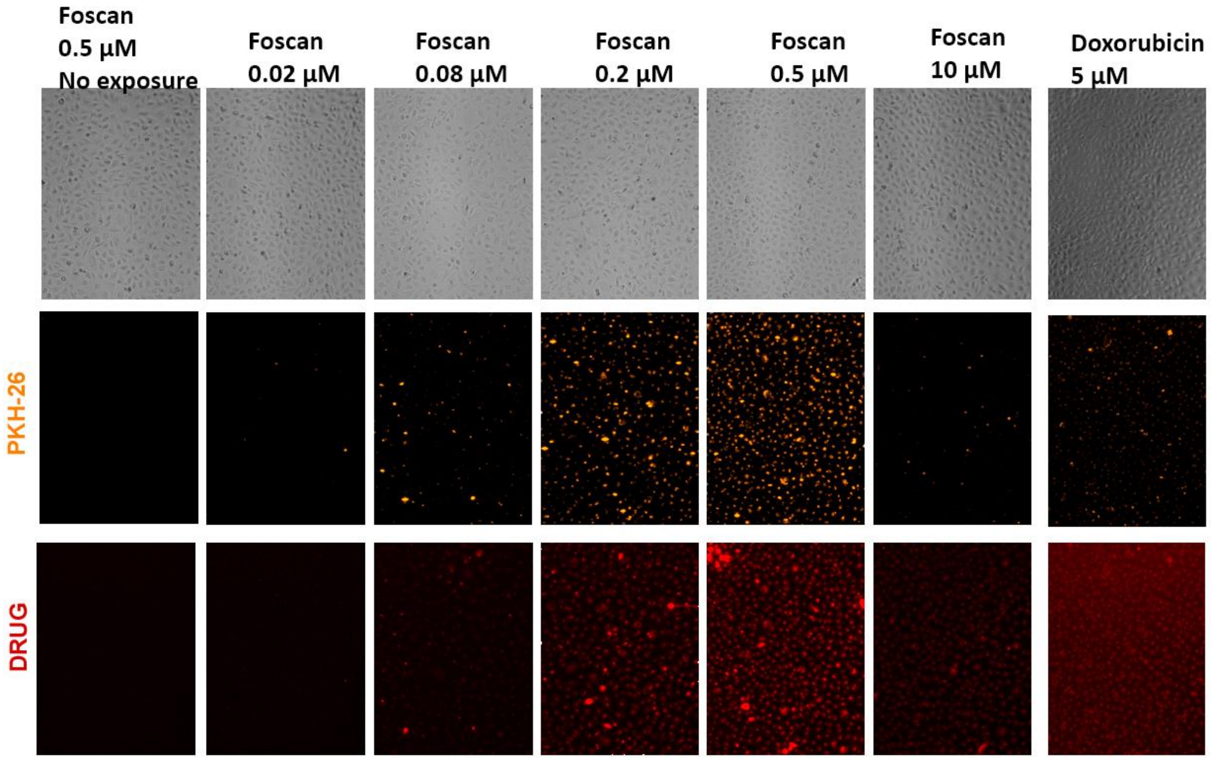

Supplementary Figure 8: Uptake of PC3 vesicles evidenced by the transfer of their membrane fluorescence staining (PKH-26) and drug load (Foscan or doxorubicin) to Huvec recipient cells by low magnification (4x) microscopy (bright field and fluorescence images). All images evidence a homogeneous vesicle uptake. Higher fluorescence emission intensity of both membrane staining and drug was observed at intermediate Foscan concentration, in agreement with vesicle counting data.

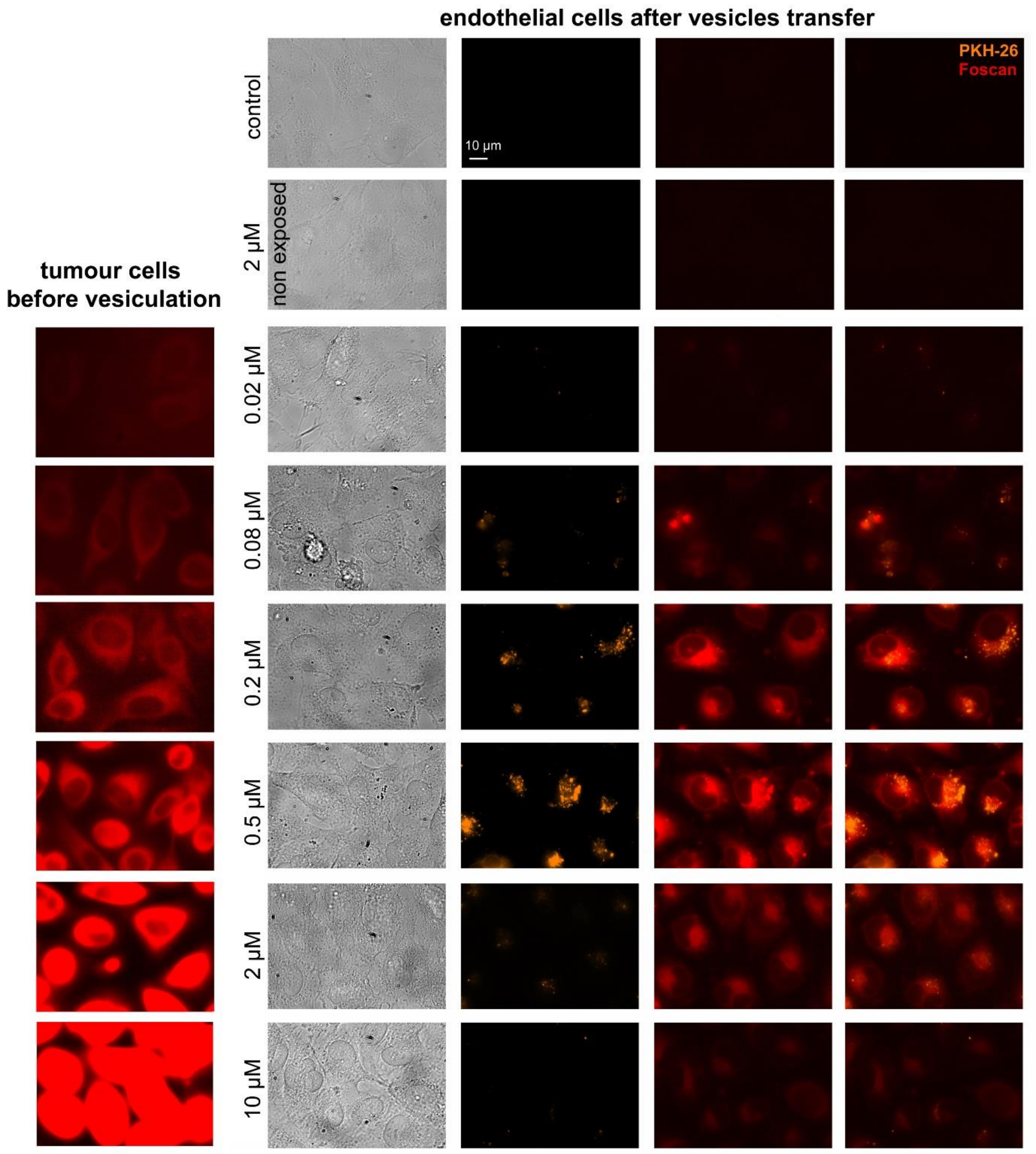

Supplementary Figure 9: Transfer of membrane fluorescence staining (PKH-26) and Foscan drug load from PC3 (first column in the left) to Huvec recipient cells (the four other columns in the center and on the right) by optical microscopy (bright field and fluorescence images with the same scaling applied to all images). Foscan fluorescence emission by PC3 donor cells increases with Foscan incubation concentration. Conversely, fluorescence emission by Huvec recipient cells was higher for both vesicle membrane staining and Foscan at

intermediate Foscan concentration, in agreement with vesicle counting data displayed at Figure 3. Quantitative transfer data are presented in Figure 4.

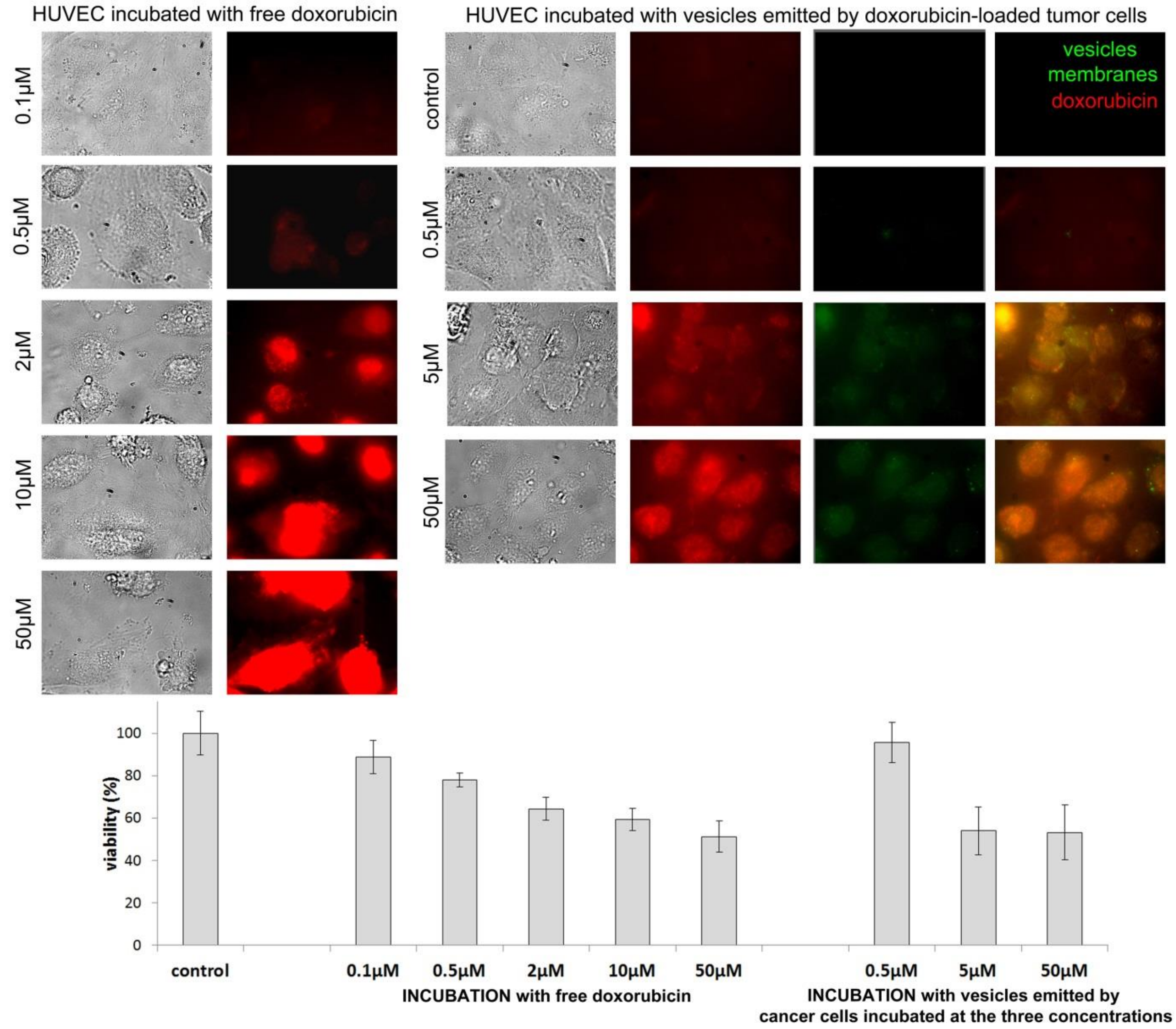

Supplementary Figure 10: Uptake of doxorubicin and viability of Huvec recipient cells after the incubation with free doxorubicin compared to the incubation with vesicles from PC3 donor cells previously submitted to doxorubicin treatment. Recipient Huvec cells incubated with free doxorubicin (two columns on the left) or vesicles from PC3 donor cells following doxorubicin treatment (four columns on the right) by optical microscopy (bright field and fluorescence images with the same scaling applied to all images). Doxorubicin fluorescence emission by Huvec cells increases with the concentration of free doxorubicin in the incubation medium. Huvec recipient cells after the incubation with vesicles from PC3 donors presented higher fluorescence emission from both vesicle membrane staining and doxorubicin at the highest doxorubicin concentration. Quantitative transfer data are presented in Figure 4. The

viability of Huvec cells incubated with free doxorubicin or vesicles was analyzed by Alamar assay showing a cytotoxic effect of vesicles following doxorubicin treatment at the highest doses (5 and 50 µM) close to the effect of the free drug at equivalent concentration ranges.
